# Neuroprognostication following out of hospital cardiac arrest - a retrospective study of departmental practice

**DOI:** 10.1186/2197-425X-3-S1-A538

**Published:** 2015-10-01

**Authors:** E Hindle, M Dunn, M Gillies, G Clegg

**Affiliations:** Intensive Care Unit, Royal Infirmary of Edinburgh, Edinburgh, United Kingdom; Emergency Department, Royal Infirmary of Edinburgh, Edinburgh, United Kingdom; University of Edinburgh, Edinburgh, United Kingdom

## Introduction

Predicting those who are likely to survive with good neurological outcome following out of hospital cardiac arrest (OHCA) is important for patients, their carers and society - however prognostication following OHCA is difficult. The European Resuscitation Council and the European Society of Intensive Care Medicine (ESICM) have published an advisory statement regarding prognostication in comatose survivors of cardiac arrest[1]. The guidelines recommend a multimodal approach, which includes somatosensory evoked potentials (SSEPs), diffusion weighted magnetic resonance imaging (MRI) sequences and a period of prolonged clinical observation in cases where prognosis is uncertain, however, some recommended investigations, for example SSEPs and MRI are inconsistently available, even in large university teaching hospitals.

## Objectives

Our objective was to investigate timing of prognostic decisions on neurological recovery after cardiac arrest and which investigations are used to aid decision-making for all patients admitted to our intensive care unit (ICU) following OHCA over a 19 month period.

## Methods

We conducted a retrospective observational study of neuroprognostication practice following OHCA in a large tertiary referral ICU. In addition to a survey of current practice, we assessed mortality in this group and neurological outcomes of survivors.

## Results

118 patients were identified for inclusion in analysis and of those notes were available for 107 (90.7%). 49.5% of patients survived to hospital discharge. The process of neuroprognostication was documented for 43 patients (79.6% of patients dying in-hospital). Investigations used to aid prognostication included: CT scanning (22 (51.2%)), EEG (3 (7.0%)). Clinical finings included: brainstem death testing (2 (4.7%)), absent pupillary reflexes (22 (51.2%)), status epilepticus (16 (37.2%)). No patient underwent magnetic resonance imaging (MRI), somatosensory evoked potentials (SSEP) or measurement of biomarkers of neurological injury.

## Conclusions

In our centre, a high proportion of survivors of OHCA survived to hospital discharge with favourable neurology, although the use of investigations to aid neuroprognistication is inconsistent and some recommended modalities are unavailable, even in a tertiary centre. Further large-scale audits are warranted to elucidate current neuroprognostication practice and availability of evidence based investigations.
